# Relationships between Nursing Students’ Skill Mastery, Test Anxiety, Self-Efficacy, and Facial Expressions: A Preliminary Observational Study

**DOI:** 10.3390/healthcare10020311

**Published:** 2022-02-07

**Authors:** Myoung Soo Kim, Byung Kwan Choi, Ju-Yeon Uhm, Jung Mi Ryu, Min Kyeong Kang, Jiwon Park

**Affiliations:** 1 Department of Nursing, Pukyong National University, Busan 48513, Korea; kanosa@pknu.ac.kr (M.S.K.); jyuhm@pknu.ac.kr (J.-Y.U.); ellekang1113@naver.com (M.K.K.); 2 Department of Neurosurgery, College of Medicine, Pusan National University Hospital, Busan 49241, Korea; spine@pusan.ac.kr; 3 Department of Nursing, Busan Institute of Science and Technology, Busan 46639, Korea; rewmis@naver.com

**Keywords:** clinical competence, test anxiety, self-efficacy, facial expression, students, nursing

## Abstract

Test anxiety and self-efficacy significantly influence the mastery of nursing skills. Facial expression recognition tools are central components to recognising these elements. This study investigated the frequent facial expressions conveyed by nursing students and examined the relationships between nursing skill mastery, test anxiety, self-efficacy, and facial expressions in a test-taking situation. Thirty-three second-year nursing students who were attending a university in a Korean metropolitan city participated. Test anxiety, self-efficacy, and facial expressions were collected while the students inserted indwelling catheters. Using Microsoft Azure software, the researchers examined the students’ facial expressions. Negative facial expressions, such as anger, disgust, sadness, and surprise, were more common during the test-taking situation than the practice trial. Fear was positively correlated with anxiety. None of the facial expressions had significant relationships with self-efficacy; however, disgust was positively associated with nursing skill mastery. The facial expressions during the practice and test-taking situations were similar; however, fear and disgust may have been indicators of test anxiety and skill mastery. To create a screening tool for detecting and caring for students’ emotions, further studies should explore students’ facial expressions that were not evaluated in this study.

## 1. Introduction

The mastery of nursing skills, which is regarded as one of the most important aspects of academic performance, is a vital component of the process of educating nursing students to ensure that they meet accountability standards for nurses [1]. A recent study of 35 countries, that were part of the Organisation for Economic Co-operation and Development, found that the factors that significantly affected academic performance were enjoyment, motivation, test anxiety, and self-efficacy [2]. Among these factors, it is widely recognised that the student’s levels of anxiety and self-efficacy when taking a skills test affects their skill mastery [3,4]. Nursing students may have higher levels of anxiety than students in other fields [5]. Based on the fact that excessive anxiety influences the acquisition and application of cognitive and psychomotor skills [6], we can predict that this could have a negative effect on skill mastery. High self-efficacy is associated with better performance in clinical skills tests [7] because skill competency requires belief in the student’s ability to use their knowledge and skills effectively. As test anxiety arises from an interaction between the cognitive and value evaluations that are related to task achievement [8], students with low self-efficacy may not believe that they can achieve successful tasks, which may also result in higher test anxiety [9,10]. Therefore, since the control of test anxiety and self-efficacy is imperative before any skills test, the importance of the early detection of these signs is clear.

Although there is significant reliance on self-reporting when examining anxiety, this method can have low sensitivity to self-reflective limitations such as the unwillingness to report on one’s own experiences [11]. To date, though no clear facial expressions have been recognised, both anxiety and self-efficacy can be identified. Moreover, few studies have measured the relationships between facial expressions, emotions, and outcomes. Facial expressions are vital features that are used to observe other people’s emotions. The face can reveal emotional states and intentions [12], and it is crucial for successful social interactions. According to the 7–38–55 rule, 7 percent of meaning is expressed through verbal communication, 38 percent through vocal communication, and 55 percent through visual communication, such as facial expressions [13]. Facial expressions convey a wide range of information; however, they are not easy to detect due to limited reliability, lack of specificity, and limited generalisability [14]. To compensate for this, computational algorithms that train machines to decipher complex associations between facial expressions and emotions have recently been developed [15].

Machine learning-based automatic facial expression recognition algorithms include functions for feature selection, classification, and extraction [16]. Particularly, the algorithm classifies emotions into a set of predefined emotional categories, showing good recognition rates of ≥80% [17]. Facial recognition algorithms are commonly designed following psychologist Paul Ekman’s model. He proposed that there are seven universal emotional expressions (i.e., happiness, sadness, surprise, fear, anger, disgust, and contempt) that can help identify emotions through these facial expressions. It is true that facial expressions can provide more detailed explanations to allow for a better understanding of emotions [17,18,19], such as anxiety or depression. In a recent study that used facial recognition technology, students who expressed more surprise and less disgust in a suicide advising session were more likely to be receptive to suicide prevention, which showed the relationship between facial expression and potential suicide prevention among students [20]. Therefore, students’ facial cues are likely to reflect their emotional state, and we propose that these characteristics can be applied to improve the quality of the education system.

If studies could identify any of the students’ facial expressions that have not yet been recognized, and if they could determine the relationships between skill mastery, test anxiety, self-efficacy, and facial expressions, this information may help alleviate students’ test anxiety and promote self-efficacy and skill mastery. Therefore, we set two research questions: (1) What is the most common facial expression extracted when using a facial recognition system in a skills test-taking situation? (2) Are there significant relationships between skill mastery, test anxiety, self-efficacy, and facial expressions in a skills test-taking situation?

## 2. Materials and Methods

### 2.1. Study Design

This study employed a prospective, observational design.

### 2.2. Settings and Participants

This study was performed from April 2021 to May 2021 and included the second-year students from one nursing school in a Korean metropolitan city. The inclusion criteria were as follows: (1) the previous completion of the ‘Fundamentals of Nursing I’ theory and practicum classes in the first semester, (2) enrolment in the 2-credit ‘Fundamentals of Nursing II’ practical course, and (3) provision of paper-based consent to participate. Students undergoing clinical practicum during the period of data collection were excluded from this research. We recruited thirty-four students in the study; however, one student was excluded because their photograph data were unavailable. The sample size was evaluated based on the largest effect size (0.50) [21], significance level (0.05), and power (0.80) using the Wilcoxon signed-rank test (matched pairs) of G*power 3.1.9.7. The required sample size was calculated to be 28 and was considered reasonable for analysis. The students’ mean age was 21.4 years old and 88% were women.

### 2.3. Instrumentation

#### 2.3.1. Nursing Skill Mastery

In this study, nursing skill mastery was defined as the ability to correctly insert an indwelling catheter. We used the checklist that was developed by the Korean Accreditation Board of Nursing and each checklist was scored by one nursing professor. This study used a two-part evaluation (skill evaluation: 60 points, overall learner preparation: 40 points). Forty steps related to the skills evaluation were assessed. The checklist included three possible outcomes for each step: ‘done correctly’, ‘done incorrectly’, and ‘not done’. The total skills evaluation score was calculated by dividing the total number of items that were performed correctly and incorrectly and multiplying that number by 60. Overall learner preparation was evaluated using the following four items: proficiency, accuracy, knowledge, and attitude/communication. The students’ scores were registered as codes in the software, with higher scores indicating greater skill mastery.

#### 2.3.2. Test Anxiety

As part of a time-saving method, anxiety was assessed using a one-item instrument. It consisted of five options for the facial expressions: smile, slight smile, neutral, slight frown, and frown. We asked the participants the following question: ‘Can you select the emoticons that best represent your levels of anxiety at present?’ The responses were assigned a range of codes (from smile = 1 to frown = 5), with higher scores indicating higher levels of anxiety. The reliability of the instrument was evaluated using the test–retest technique to determine the consistency between two different completion times. The correlation was 0.88.

#### 2.3.3. Self-Efficacy

We used the 28-items instrument that was developed by Kim and Park to test self-efficacy [22]. It included three subcategories: confidence, self-control efficacy, and preference regarding the assignment’s difficulty levels (8, 10, and 10 items, respectively). All the questions were answered using a six-point Likert scale (ranging from 1 = strongly disagree to 6 = strongly agree), with higher scores indicating higher self-efficacy levels. In this study, Cronbach’s α was 0.70.

#### 2.3.4. Facial Expression Recognition

**Figure 1:**
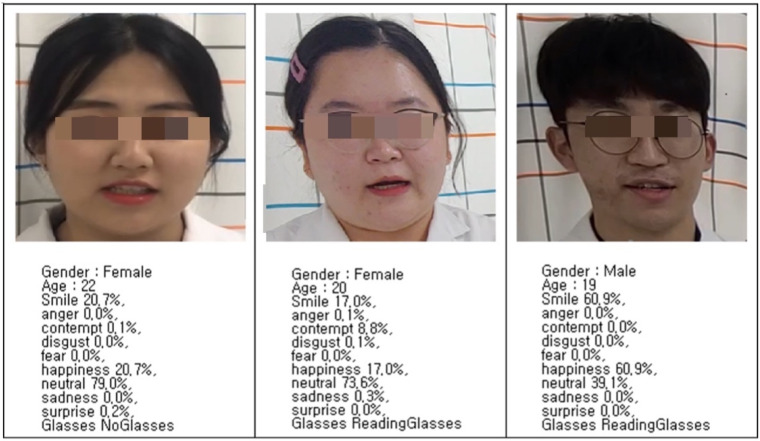
Example of facial expression analysis.

Azure (Microsoft, Redmond, WA, USA) was used to recognise the participants’ facial expressions. It is a database that is based on machine learning algorithms that use face detection to extract face-related attributes, verify whether two faces belong to the same person, find similar faces, and group faces. The database comprises the facial expressions of eight basic emotions: anger, contempt, disgust, fear, happiness, neutrality, sadness, and surprise. It provides face detection and classification. Images of facial expressions are classified into two categories, standardised and non-standardised. Standardised images are a collection of images created in the lab (i.e., fixed lighting, head positions, and view). Non-standardised images stem from more natural settings (i.e., stills from movie scenes that display emotions in the actors’ faces) [23]. Azure showed superior performance on non-standardised facial expressions among other facial expression recognition tools [24] that performed facial expression analysis. When we input one snapshot in the application, it showed the facial emotion analysis and saved the data to an Excel Spreadsheet (Microsoft, Redmond, WA, USA). Figure 1 shows the analysis results of Azure. Age was estimated. Azure has a stable age-related error [25], therefore, we did not use this result.

### 2.4. Data Collection

Data collection was performed twice. First, in a practice class, we checked the anxiety and self-efficacy of each participant. In addition, participants were advised to submit their video clips using their own smartphone during the practice run to allow for the further gathering of facial expression recognition data. They were instructed to create a video clip by placing their smartphone directly in front of themselves and focusing directly on their faces. Second, we assessed skill mastery, anxiety, and self-efficacy in the test-taking situation. During the skills tests, we placed an action camera (GoPro Hero 8; GoPro, San Mateo, CA, USA) with a resolution of 640 × 480 pixels to similarly create another video. Thereafter, one of the researchers extracted two to three snapshots from the practice class video clips and four or five snapshots from the skill test clips.

### 2.5. Ethical Considerations

This study was approved by the Institutional Review Board (No: 1041386-202103-HR-6-01) from the affiliate organisation of researchers. During the recruitment period, the study purpose, research methods, voluntary participation, guarantee of anonymity for research participation, and capability to withdraw from participation were explained to the participants in detail. Written consent was obtained from the participants prior to their involvement in the study. The participants received a coffee coupon (worth 20 USD) as compensation.

### 2.6. Data Analysis

We used IBM SPSS statistical software version 25.0 (IBM Corp., Armonk, NY, USA) and used descriptive statistics to define nursing skill mastery, test anxiety, self-efficacy, and facial expressions. To analyse the facial expressions, 96 and 157 photographs from the practice class and test-taking situation, respectively, were converted into the 33 participants’ data from the two situations. We used the percentage for each facial expression emotion, not the category, since our data were non-standardised expressions and did not classify specific facial categories. Facial expression emotions were coded with the percentage of each emotion. The Wilcoxon signed-rank test was used to compare the differences between the eight subcategory facial expressions in the practice class and skills test-taking situation. Spearman’ Rank Correlation(s) was conducted to investigate the correlation between the variables.

## 3. Results

**Figure 2:**
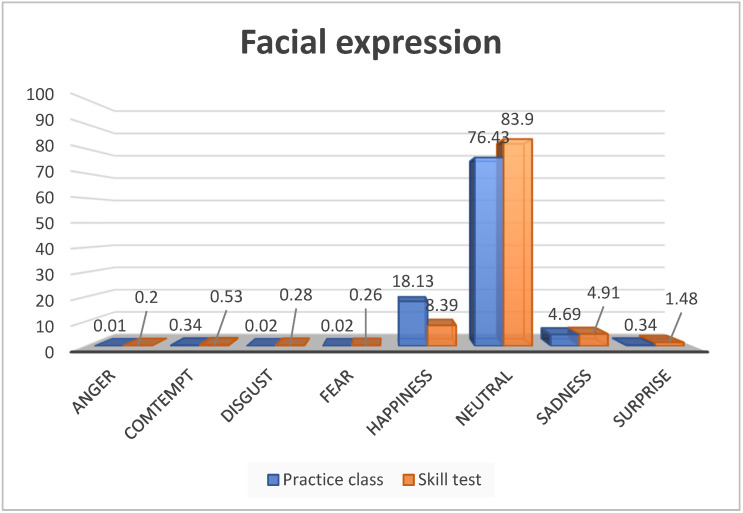
Comparison graph of facial expression subcategories.

The age of the participants ranged from 21 to 24 (mean age = 22.03, SD = 1.37). Of the total 33 participants, 87.9% were female. Figure 2 shows the mean proportion of each facial expression. In both the practice class and skills test-taking situation, the most frequent facial expressions were neutral and happy. Negative facial expressions, such as anger (Z = 3.70, *p* < 0.001), disgust (Z = 3.39, *p* = 0.001), sadness (Z = 2.46, *p* = 0.014), and surprise (Z = 2.55, *p* = 0.011), were significantly more common in the test-taking situation than in the practice class. We observed a significant difference in the anxiety level when the two periods were compared (Z = 2.83, *p* = 0.005); however, there was no significant difference in self-efficacy (Z = 0.88, *p* = 0.379) (Table 1).

Fear was positively correlated with anxiety (rs = 0.35, *p* = 0.046); however, none of the facial expressions were significantly associated with self-efficacy (Table 2). Disgust was positively associated with nursing skill mastery (rs = 0.36, *p* = 0.042).

## 4. Discussion

Automatic facial expression recognition can be used in patient care [26] and medical education [27], as well as in virtual reality [28] and augmented reality-based games [29]. Although some studies have explored the link between facial expression recognition technology and academic performance [18,19,30], there is limited research in the field of nursing education. The establishment of this information could serve as a supplementary screening tool for improving patient assessments [26]. Moreover, it will be helpful with nursing education as it allows for the detection and care of students’ emotions. For these reasons, we focused on the results of the research questions.

In this study, there were no conspicuous types of expressions. We did not make a standardised photograph frame for more naturalistic and spontaneous facial expressions. Therefore, acquiring prototypical facial expressions is difficult since non-standardised photographs contain large variations in the expression itself, circumstances, and a person’s characteristics. During the two situations, students mostly demonstrated neutral or happy facial expressions. It is in line with more neutral ratings for non-standardised photographs compared to standardised [31]. Therefore, we focused on the comparison between the two situations.

Negative facial expressions including anger, disgust, and sadness were significantly more frequent in the test-taking situation compared to the practice class. The students may have been afraid of receiving negative evaluations, losing scholarships, and being prevented from becoming a nurse due to their poor skills [32,33]. These high-stakes situations may have evoked negative emotions during the exam, which could have resulted in the students feeling fearful or anxious [32]. The act of reading people’s emotions can prove difficult when the individuals are not actively using their faces to express themselves. Furthermore, caution should be exercised when assessing facial expressions as there may be bias present depending on the participant’s age, sex, and race. For example, studies demonstrated that older adult faces were believed to convey more negative emotions due to the presence of age-related features, such as a downturned mouth [34]; structurally, women’s faces resembled happy faces [35]; and White people’s faces were perceived to convey angrier expressions than African American or Korean people’s faces [36]. The use of automatic facial expression recognition software in nursing education can help identify when a student is experiencing a tense emotional situation and, subsequently, help them avoid taking a test in a tense situation. Therefore, we propose that prominent expressions should be identified through repeated research.

Nursing skill mastery was only positively correlated with facial expression of disgust. This finding was in line with previous study results when people concentrated on something that could be perceived as a frown, due to the wrinkling of their foreheads and furrowing of their brows, which was similar to the expression of disgust [37]. Because immersive experiences successfully increased the students’ confidence and nursing skills [38], we considered the association between the expression of disgust and achieving nursing skill mastery as a result of immersive test-taking. Considering that recognising emotions through facial expressions during the test-taking process plays an important role in supporting better academic performance through effective regulation of the students’ emotions [39], educators should take better care of students’ emotions. According to a recent study, complementary and alternative methods that were implemented by educators were effective in relieving test anxiety [40] and improving performance [41]. Therefore, it is imperative which interventions could help students effectively cope and could also be applied during classes.

Anxiety was significantly associated with the facial expressions related to fear. This could be called test anxiety because it increased significantly during the skills test-taking situation. Anxiety is related to passive avoidance behaviours and fear is related to active avoidance behaviours [42], although these states are not detectable through psychometric means [43]. The results from this study demonstrated that facial expressions that depicted anxiety and fear were similar. Because anxiety is considered a composite feeling that is mainly affected by fear, when a person experiences anxiety, one can expect to see facial signs that depict fear. Furthermore, although the facial signs of anxiety are ambiguous and study results are inconsistent, anxiety’s effects on the face are thought to be related to alterations in eye movements, skin reddening, and lip distortions [44]. Anxiety is a critical emotion when considered in terms of individual differences in facial expression recognition [45]. However, if anxiety cannot be accurately detected in the face, fearful facial expressions may be regarded as a representation of anxiety. Therefore, the fact that fearful expressions were prominent in the skills test-taking situation indicated that fear could be used as part of a screening tool for students’ emotions by incorporating it with other indicators.

Self-efficacy was not associated with any of the facial expressions, and this meant that judging self-efficacy based on facial expressions could prove difficult. There are two plausible explanations. First, we propose that we may not have observed a significant relationship between self-efficacy and facial expression because the students did not outwardly express their emotions in the test-taking situation. Second, students’ anxiety levels could have been so high [5] that they dominated their capacity for self-efficacy; thus, self-efficacy could not be inferred from their facial expressions. Furthermore, in this study, self-efficacy was not related to anxiety or nursing skill mastery, which was inconsistent with the findings from other studies. Self-efficacy may have protected the students from the negative effects of anxiety with respect to their academic performance [46], and may have partially mediated the relationship, which demonstrated that higher stress levels affected poorer academic performance during exams [47]. Particularly, higher self-efficacy was positively correlated with nursing students correctly performing cardiac compression skills [48], catheter care, and infiltration monitoring [49]. Although whether the emotions are negative or positive during specific situations does not completely affect the outcomes, the ways in which students manage their emotions could have an impact on the outcomes [50]. Therefore, the preparation of emotional management through facial expression research will prove helpful in improving nursing students’ skill mastery.

It is necessary to consider whether facial expression emotions could generate similar high neutral rates if other topics, such as intravenous injection, were selected. We believe the same results would be revealed for two reasons aside from the fact that non-standardised photographs mostly demonstrated neutrality. First, indwelling foley catheterisation is one of the most challenging and meticulous nursing procedures with similar skill requirements as for a venous puncture [51]. Both skills involve complex clinical procedures using aseptic techniques. As a result, students demonstrate similar confidence in performing intravenous injections and indwelling urinary catheterisation than any other basic nursing procedure [52,53]. Second, students reach a high concentration level and focus on their job during the skill test. According to a recent study, students display a neutral emotion pose in a high concentration situation [54]. Therefore, further investigation, including tracking eye movement and emotion-specificity is needed because it is difficult to distinguish between students’ anxiety and neutrality.

Since the onset of the COVID-19 outbreak, wearing masks has become the new normal and this practice has had a pervasive influence on daily life. Because facial emotion recognition relies on the ability to view all aspects of the face, including the nose, mouth, and jaw, the accuracy of the technology may be reduced when only the periorbital area that is not covered by a mask is analysed [55]. To overcome the challenge of mask-wearing in relation to facial emotion recognition, more refined facial recognition programs should be developed [56]. However, we recognise that, even at the current level of technology, if students’ expressions can be captured and read in online test situations, these systems can be used more frequently than face-to-face tests; thus, ultimately, assisting with caring for students’ emotions.

## 5. Study Strengths and Limitations

The strength of this study is that it contributes significantly to the existing literature, as it sheds light on the use of facial emotion recognition technology in nursing education. If further studies can deduce the typical types of facial expressions conveyed by students during test-taking situations, this information can be used as basic data that can improve nursing skill mastery by reducing the anxiety of students and improving their confidence.

However, this study has some limitations. First, the sample size was small. A multi-institution study could not be conducted due to limited face-to-face classes during the COVID-19 pandemic. Furthermore, static features can be insufficient for precisely analysing students’ facial emotions utilised in this study. To generalise the facial expression results, future researchers need to utilise video-based sequences to obtain dynamic information and increase facial emotion recognition [57,58]. Second, because a limited number of variables were included in this study, other factors (including skills test-related emotional factors such as passion, a feeling of achievement, disappointment, and dissatisfaction) were not checked. Third, owing to the differences in the video recording tools used in the practice class and test-taking situations, interpretation bias could have been introduced because of the differences in resolution. When comparing the facial expressions, our visual system had lower perceptual tolerance for processing ambiguous expressions that exhibited conditional interpretation bias [59]. Fourth, we had the challenge of getting clear grouped data of images captured in video clips since they are taken in non-standardised settings. To cope with this challenge, the use of facial landmarks alongside the original image is helpful [60]. It can act as an embedded regulation that can weigh facial expressions in the classification of emotions during skill-test situations. Fifth, we did not consider how gender could influence the research (female nurses with male patients, and vice versa). Performing urinary catheterisation on a patient of the opposite gender may influence care providers’ emotions, self-efficacy, and skill mastery, regardless of their urethral knowledge [61,62]. Future research needs to explore the potential differences among nursing students when it comes to providing sensitive care to patients of the opposite gender. Cultural differences can greatly influence genital-related care, including urinary catheterisation, and should be investigated in future studies [63,64,65]. Therefore, we propose that, in comparison to our study, future studies should employ a larger sample size, more discursive factors, better congruency between the video resolutions, and the use of facial landmarks to rectify these limitations.

## 6. Conclusions

In the skills test-taking situation, neutral and happy expressions were seen the most frequently; however, these findings were similar to those of the practice class and no dominant types of expressions were identified. Fear and disgust could be representative expressions for test anxiety and self-efficacy, respectively. Further studies should explore other facial expressions based on the students’ emotions and should adopt facial emotion recognition technology to care for the significant emotional stress that students endure.

## Figures and Tables

**Table 1:** Comparison of test anxiety, academic efficacy, and facial expression between practice class and skill test (*n* = 33).

Variables	Practice Class	Skill Test	Z (*p*)
	M ± SD		
Test anxiety	2.00 ± 0.85	2.52 ± 0.87	2.83 (0.005)
Self-efficacy	3.66 ± 0.37	3.69 ± 0.41	0.88 (0.379)
Facial expression (anger)	0.01 ± 0.04	0.20 ± 0.45	3.70 (<0.001)
Facial expression (contempt)	0.34 ± 1.51	0.53 ± 0.98	1.94 (0.052)
Facial expression (disgust)	0.02 ± 0.05	0.28 ± 0.89	3.39 (0.001)
Facial expression (fear)	0.01 ± 0.03	0.26 ± 1.10	1.85 (0.064)
Facial expression (happiness)	18.13 ± 28.02	8.39 ± 14.04	1.72 (0.085)
Facial expression (neutral)	76.43 ± 29.88	83.90 ± 19.20	1.26 (0.208)
Facial expression (sadness)	4.69 ± 15.36	4.91 ± 5.52	2.46 (0.014)
Facial expression (surprise)	0.34 ± 0.65	1.48 ± 4.11	2.55 (0.011)

**Table 2:** Correlations between nursing skill mastery, test anxiety, academic self-efficacy, and facial expressions (*n* = 33).

	1	2	3	4	5	6	7	8	9	10
	rs									
1. Nursing skill mastery	1.00									
2. Test anxiety	0.02 (0.933)	1.00								
3. Self-efficacy	0.29 (0.103)	−0.01 (0.995)	1.00							
4. Facial expression (anger)	0.17 (0.349)	0.05 (0.780)	0.01 (0.945)	1.00						
5. Facial expression (contempt)	−0.13 (0.471)	0.17 (0.354)	−0.10 (0.587)	−0.09 (0.639)	1.00					
6. Facial expression (disgust)	0.36 (0.042)	0.07 (0.712)	−0.18 (0.306)	0.53 (0.002)	0.04 (0.828)	1.00				
7. Facial expression (fear)	0.20 (0.264)	0.35 (0.046)	0.20 (0.257)	0.11 (0.548)	0.30 (0.086)	0.05 (0.775)	1.00			
8. Facial expression (happiness)	0.05 (0.770)	−0.25 (0.168)	−0.20 (0.263)	−0.17 (0.355)	0.30 (0.088)	0.40 (0.022)	0.21 (0.213)	1.00		
9. Facial expression (neutral)	−0.01 (0.946)	0.23 (0.201)	0.15 (0.394)	−0.04 (0.810)	−0.33 (0.058)	−0.43 (0.012)	−0.34 (0.052)	−0.77 (<0.001)	1.00	
10. Facial expression (sadness)	−0.06 (0.731)	−0.05 (0.765)	−0.02 (0.896)	0.37 (0.034)	0.21 (0.250)	0.31 (0.076)	0.41 (0.019)	0.20 (0.268)	−0.57 (0.001)	1.00
11. Facial expression (surprise)	0.11 (0.543)	0.24 (0.170)	0.25 (0.168)	−0.08 (0.660)	0.06 (0.753)	−0.08 (0.653)	0.68 (<0.001)	0.27 (0.119)	−0.27 (0.126)	0.07 (0.719)

## Data Availability

The datasets generated and analysed during the current study are not publicly available but are available from the corresponding author upon reasonable request.

## References

[1] Shen L., Zeng H., Jin X., Yang J., Shang S., Zhang Y. (2018). An innovative evaluation in fundamental nursing curriculum for novice nursing students: An observational research. J. Prof. Nurs..

[2] Govorova E., Benítez I., Muñiz J. (2020). Predicting Student Well-Being: Network Analysis Based on PISA 2018. Int. J. Environ. Res. Public Health.

[3] Wu J.H., Du J.K., Lee C.Y., Lee H.E., Tsai T.C. (2020). Effects of anxiety on dental students’ noncognitive performance in their first objective structured clinical examination. Kaohsiung J. Med. Sci..

[4] Terzi B., Daş Geçim G.Y., Topuz İ. (2019). Identification of the self-confidence and self-efficacy levels of student nurses when performing blood drawing for the first time on their peers. Technol. Health Care Off. J. Eur. Soc. Eng. Med..

[5] Bartlett M.L., Taylor H., Nelson J.D. (2016). Comparison of Mental Health Characteristics and Stress Between Baccalaureate Nursing Students and Non-Nursing Students. J. Nurs. Educ..

[6] Dorison C.A., Klusowski J., Han S., Lerner J.S. (2020). Emotion in organizational judgment and decision making. Organ. Dyn..

[7] Jones A., Sheppard L. (2011). Self-efficacy and clinical performance: A physiotherapy example. Adv. Physiother..

[8] Pekrun R. (2006). The Control-Value Theory of Achievement Emotions: Assumptions, Corollaries, and Implications for Educational Research and Practice. Educ. Psychol. Rev..

[9] Bandura A. (1978). Self-efficacy: Toward a unifying theory of behavioral change. Adv. Behav. Res. Ther..

[10] Krispenz A., Gort C., Schültke L., Dickhäuser O. (2019). How to Reduce Test Anxiety and Academic Procrastination Through Inquiry of Cognitive Appraisals: A Pilot Study Investigating the Role of Academic Self-Efficacy. Frotiers Psychol..

[11] Ganellen R.J. (2007). Assessing normal and abnormal personality functioning: Strengths and weaknesses of self-report, observer, and performance-based methods. J. Personal. Assess..

[12] Jain D.K., Shamsolmoali P., Sehdev P. (2019). Extended deep neural network for facial emotion recognition. Pattern Recognit. Lett..

[13] Mehrabian A. (2007). Nonverbal Communication.

[14] Barrett L.F., Adolphs R., Marsella S., Martinez A.M., Pollak S.D. (2019). Emotional Expressions Reconsidered: Challenges to Inferring Emotion from Human Facial Movements. Psychol. Sci. Public Interest: A J. Am. Psychol. Soc..

[15] Kim J.H., Poulose A., Han D.S. (2021). The Extensive Usage of the Facial Image Threshing Machine for Facial Emotion Recognition Performance. Sensors.

[16] Murugappan M., Mutawa A. (2021). Facial geometric feature extraction based emotional expression classification using machine learning algorithms. PLoS ONE.

[17] Yamaguchi N., Caceres M.N., De la Prieta F., Matsui K. (2016). Facial Expression Recognition System for User Preference Extraction. Distributed Computing and Artificial Intelligence, 13th International Conference (DCAI 2016).

[18] Santos P.B., Wahle C.V., Gurevych I. Using Facial Expressions of Students for Detecting Levels of Intrinsic Motivation. Proceedings of the 2018 IEEE 14th International Conference on e-Science (e-Science).

[19] Mohamad Nezami O., Dras M., Hamey L., Richards D., Wan S., Paris C. (2020). Automatic Recognition of Student Engagement Using Deep Learning and Facial Expression.

[20] Hu C.S., Ji J., Huang J., Feng Z., Xie D., Li M., Liang Z., Wei Z. (2020). Wiser Reasoning and Less Disgust Have the Potential to Better Achieve Suicide Prevention. Crisis.

[21] Ricciardi L., Visco-Comandini F., Erro R., Morgante F., Bologna M., Fasano A., Ricciardi D., Edwards M.J., Kilner J. (2017). Facial Emotion Recognition and Expression in Parkinson’s Disease: An Emotional Mirror Mechanism?. PLoS ONE.

[22] Kim A., Park I. (2001). Construction and validation of academic self-efficacy scale. Korean J. Educ. Psychol..

[23] Dhall A., Kaur A., Goecke R., Gedeon T. EmotiW 2018: Audio-Video, Student Engagement and Group-Level Affect Prediction. Proceedings of the 20th ACM International Conference on Multimodal Interaction.

[24] Küntzler T., Höfling T.T.A., Alpers G.W. (2021). Automatic Facial Expression Recognition in Standardized and Non-standardized Emotional Expressions. Front. Psychol..

[25] Jung S.-G., An J., Kwak H., Salminen J., Jansen B.J. Assessing the accuracy of four popular face recognition tools for inferring gender, age, and race. Proceedings of the Twelfth international AAAI Conference on Web and Social Media (ICWSM 2018).

[26] Chen L., Ma X., Zhu N., Xue H., Zeng H., Chen H., Wang X. (2021). Facial Expression Recognition with Machine Learning and Assessment of Distress in Patients With Cancer. Oncol. Nurs. Forum.

[27] Marwaha A., Chitayat D., Meyn M.S., Mendoza-Londono R., Chad L. (2021). The point-of-care use of a facial phenotyping tool in the genetics clinic: Enhancing diagnosis and education with machine learning. Am. J. Med. Genet. Part A.

[28] Bekele E., Bian D., Peterman J., Park S., Sarkar N. (2017). Design of a Virtual Reality System for Affect Analysis in Facial Expressions (VR-SAAFE); Application to Schizophrenia. IEEE Trans. Neural Syst. Rehabil. Eng. A Publ. IEEE Eng. Med. Biol. Soc..

[29] Hbali Y., Ballihi L., Sadgal M., El Fazziki A. (2016). Face Detection for Augmented Reality Application Using Boosting-based Techniques. Int. J. Interact. Multimed. Artif. Intell..

[30] Chiu M.-H., Liaw H.L., Yu Y.-R., Chou C.-C. (2019). Facial micro-expression states as an indicator for conceptual change in students’ understanding of air pressure and boiling points. Br. J. Educ. Technol..

[31] Dupré D., Krumhuber E.G., Küster D., McKeown G.J. (2020). A performance comparison of eight commercially available automatic classifiers for facial affect recognition. PLoS ONE.

[32] Ramirez G., Beilock S.L. (2011). Writing about testing worries boosts exam performance in the classroom. Science.

[33] Quinn B.L., Peters A. (2017). Strategies to Reduce Nursing Student Test Anxiety: A Literature Review. J. Nurs. Educ..

[34] Hess U., Adams R.B., Simard A., Stevenson M.T., Kleck R.E. (2012). Smiling and sad wrinkles: Age-related changes in the face and the perception of emotions and intentions. J. Exp. Soc. Psychol..

[35] Adams R.B., Hess U., Kleck R.E. (2014). The Intersection of Gender-Related Facial Appearance and Facial Displays of Emotion. Emot. Rev..

[36] Zebrowitz L.A., Kikuchi M., Fellous J.-M. (2010). Facial resemblance to emotions: Group differences, impression effects, and race stereotypes. J. Personal. Soc. Psychol..

[37] Lee I., Jung H., Ahn C.H., Seo J., Kim J., Kwon O. (2016). Real-time personalized facial expression recognition system based on deep learning. 2016 IEEE International Conference on Consumer Electronics (ICCE).

[38] Fowler S.M., Knowlton M.C., Putnam A.W. (2018). Reforming the undergraduate nursing clinical curriculum through clinical immersion: A literature review. Nurse Educ. Pract..

[39] Harley J.M., Pekrun R., Taxer J.L., Gross J.J. (2019). Emotion regulation in achievement situations: An integrated model. Educ. Psychol..

[40] Hopkins K. (2018). Aromatherapy for test anxiety in college students. J. Contemp. Chiropr..

[41] Son H.K., So W.-Y., Kim M. (2019). Effects of aromatherapy combined with music therapy on anxiety, stress, and fundamental nursing skills in nursing students: A randomized controlled trial. Int. J. Environ. Res. Public Health.

[42] McNaughton N., Gray J.A. (2000). Anxiolytic action on the behavioural inhibition system implies multiple types of arousal contribute to anxiety. J. Affect. Disord..

[43] Perkins A.M., Inchley-Mort S.L., Pickering A.D., Corr P.J., Burgess A.P. (2012). A facial expression for anxiety. Comp. Study.

[44] Claudino R.G.E., de Lima L.K.S., de Assis E.D.B., Torro N. (2019). Facial expressions and eye tracking in individuals with social anxiety disorder: A systematic review. Psicol. Reflexão E Crítica Psycology Res. Rev..

[45] Palermo R., Jeffery L., Lewandowsky J., Fiorentini C., Irons J.L., Dawel A., Burton N., McKone E., Rhodes G. (2018). Adaptive face coding contributes to individual differences in facial expression recognition independently of affective factors. J. Exp. Psychol. Hum. Percept. Perform..

[46] Ferreira É.d.M.R., Pinto R.Z., Arantes P.M.M., Vieira É.L.M., Teixeira A.L., Ferreira F.R., Vaz D.V. (2020). Stress, anxiety, self-efficacy, and the meanings that physical therapy students attribute to their experience with an objective structured clinical examination. BMC Med. Educ..

[47] Crego A., Carrillo-Diaz M., Armfield J.M., Romero M. (2016). Stress and Academic Performance in Dental Students: The Role of Coping Strategies and Examination-Related Self-Efficacy. J. Dent. Educ..

[48] Roh Y.S., Issenberg S.B. (2014). Association of cardiopulmonary resuscitation psychomotor skills with knowledge and self-efficacy in nursing students. Int. J. Nurs. Pract..

[49] Dogu Kokcu O., Cevik C. (2020). The Predictive Strength of Students’ Self-Efficacy, Problem Solving Skills to Perform Catheter Care. J. Korean Acad. Nurs..

[50] Finney S.J., Perkins B.A., Satkus P. (2020). Examining the simultaneous change in emotions during a test: Relations with expended effort and test performance. Int. J. Test..

[51] Korean Accreditation Board of Nursing Education (2017). Evaluation Core Basic Nursing Skill Items. 4.1 Ed. Korean Accreditation Board of Nursing Education: Seoul. http://old.kabone.or.kr/HyAdmin/upload/goodFile/120180126142812.pdf.

[52] Han A., Cho D.S., Won J. (2014). A study on learning experiences and self-confidence of core nursing skills in nursing practicum among final year nursing students. J. Korean Acad. Fundam. Nurs..

[53] Kim Y.-H., Hwang S.Y., Lee A.-Y.I. (2014). Perceived confidence in practice of core basic nursing skills of new graduate nurses. J. Korean Acad. Soc. Nurs. Educ..

[54] Sharma P., Joshi S., Gautam S., Maharjan S., Filipe V., Reis M.J. (2019). Student engagement detection using emotion analysis, eye tracking and head movement with machine learning. arXiv preprint.

[55] Libby C., Ehrenfeld J. (2021). Facial Recognition Technology in 2021: Masks, Bias, and the Future of Healthcare. J. Med. Syst..

[56] Marini M., Ansani A., Paglieri F., Caruana F., Viola M. (2021). The impact of facemasks on emotion recognition, trust attribution and re-identification. Sci. Rep..

[57] Ko B.C. (2018). A brief review of facial emotion recognition based on visual information. Sensors.

[58] Liu D., Zhang H., Zhou P. (2021). Video-based facial expression recognition using graph convolutional networks. 2020 25th International Conference on Pattern Recognition (ICPR).

[59] Kinchella J., Guo K. (2021). Facial Expression Ambiguity and Face Image Quality Affect Differently on Expression Interpretation Bias. Perception.

[60] Almeida J., Vilaça L., Teixeira I.N., Viana P. (2021). Emotion Identification in Movies through Facial Expression Recognition. Appl. Sci..

[61] Yang G., Liu H., Wang J., Geng Z., Wang L., Xu T. (2021). Genitalia-related nursing embarrassment and its associated factors among female nurses in mainland China: A nationwide cross-sectional study. Ann. Transl. Med..

[62] Zang Y.-L., Chung L.Y.F., Wong T.K.S., Chan M.F. (2009). Chinese female nurses’ perceptions of male genitalia-related care—Part 2. J. Clin. Nurs..

[63] Shallcross P. (2000). Male catheterization and the extended role of the female nurse. Br. J. Community Nurs..

[64] Kouta C., Kaite C.P. (2011). Gender discrimination and nursing: A Literature review. J. Prof. Nurs..

[65] Yip Y.-C., Yip K.-H., Tsui W.-K. (2021). Exploring the gender-related perceptions of male nursing students in clinical placement in the Asian context: A qualitative Study. Nurs. Rep..

